# Prehospital video triage of suspected stroke patients in Greater Manchester: pilot project report

**DOI:** 10.1136/bmjoq-2024-002954

**Published:** 2025-01-02

**Authors:** Ibrahim Alghamdi, Lisa Brunton, Christopher Ashton, David A Jenkins, Adrian R Parry-Jones

**Affiliations:** 1 Division of Cardiovascular Sciences, The University of Manchester, Manchester, UK; 2 Department of Emergency Medical Services, College of Applied Medical Sciences, Khamis Mushait Campus, King Khalid University, Abha, Asir, Saudi Arabia; 3 School of Health Sciences, The University of Manchester Division of Population Health Health Services Research and Primary Care, Manchester, UK; 4 Greater Manchester Neurorehabilitation & Integrated Stroke Delivery Network, Northern Care Alliance NHS Foundation Trust, Manchester, UK; 5 Division of Informatics Imaging and Data Sciences, The University of Manchester, Manchester, UK; 6 Geoffrey Jefferson Brain Research Centre, Faculty of Biology, Medicine and Health, The University of Manchester, Manchester, UK

**Keywords:** Quality improvement, Prehospital care, Telemedicine

## Abstract

**Introduction:**

Stroke is a leading cause of mortality and morbidity, demanding prompt and accurate identification. However, prehospital diagnosis is challenging, with up to 50% of suspected strokes having other diagnoses. A prehospital video triage (PHVT) system was piloted in Greater Manchester to improve prehospital diagnostic accuracy and appropriate conveyance decisions.

**Method:**

A service evaluation of a PHVT pilot was conducted to assess PHVT efficacy and identify facilitators and barriers. The pilot (October–December 2022) was a collaboration between the North West Ambulance Service, Greater Manchester Neurorehabilitation and Integrated Stroke Delivery Network and stroke clinicians at Salford Royal Hospital. The service evaluation was mixed methods, including qualitative semistructured interviews with stroke clinicians, paramedics and patients (and/or caregivers). Interviews were analysed using a thematic approach.

**Results:**

Out of 46 PHVT calls during the pilot, eight (18%) were diverted to local emergency department, 1 (2%) was left at their usual residence and 37 (80%) were transported to Salford Royal Hospital. Final diagnosis for PHVT patients was stroke in 15 (33%) of cases, non-stroke in 20 (43%) and transient ischaemic attack in 11 (24%).

Patients/caregivers found PHVT beneficial in directing them to appropriate hospitals and streamlining admission and treatment. However, some reported delays as a result. Clinicians expressed mixed opinions regarding PHVT’s utility. Paramedics found PHVT improved confidence in managing stroke patients. Hospital clinicians believed it provided valuable prearrival patient information, enhancing preparation. Others found PHVT less effective due to on-scene delays, challenges conducting comprehensive assessments over video and inability to identify all non-stroke cases.

**Conclusion:**

PHVT was viewed favourably by most patients for enhancing the care quality. Paramedics and hospital clinicians acknowledge PHVT’s support in making appropriate conveyance decisions and improving the preparation process before the patient’s arrival. Participants, however, suggested prearrival registration, 24-hour availability and clinicians' buy-in for a more effective future rollout.

**WHAT IS ALREADY KNOWN ON THIS TOPIC**

- Despite the importance of accurate stroke identification in the prehospital setting, it remains challenging due to various factors. There have been multiple attempts to enhance stroke detection, however, misdiagnosis continues to be an issue.

**WHAT THIS STUDY ADDS**

- A prehospital video triage (PHVT) system can improve quality of care, assist prehospital clinicians with stroke management, and provide hospital clinicians with more detailed information before the patient’s arrival.

**HOW THIS STUDY MIGHT AFFECT RESEARCH, PRACTICE OR POLICY**

- This evaluation shows that the PHVT system has the potential to enhance prehospital stroke detection, provide assistance in transporting patients to the appropriate hospital and facilitate treatment on arrival. Effective integration with the hospital system, proper training and wider awareness and buy-in from healthcare providers are essential.

## Problem and background

 Stroke is one of the leading causes of death and disability that may require rapid treatment for some of its specific subgroups,^1 2^ including intravenous thrombolysis (IVT) for selected ischaemic strokes^3 4^ and mechanical thrombectomy (MT) for large vessel occlusion (LVO).^5 6^ These treatments are highly time-sensitive in terms of their efficacy. IVT is most effective when administered within a 4.5-hour window, with earlier intervention strongly correlating with better outcomes.^5 7^ MT shows the greatest benefit when initiated within 6 hours.^8 9^ Therefore, timely recognition of stroke and prompt treatment with IVT or MT can greatly improve patient outcomes, reducing brain injury and minimising long-term disability.^10 11^ However, prehospital stroke diagnosis is challenging, with 30%–50% of suspected cases having other diagnoses (stroke mimics), due to the limited specificity of existing diagnostic instruments.^12^,^14^

**Figure 1:**
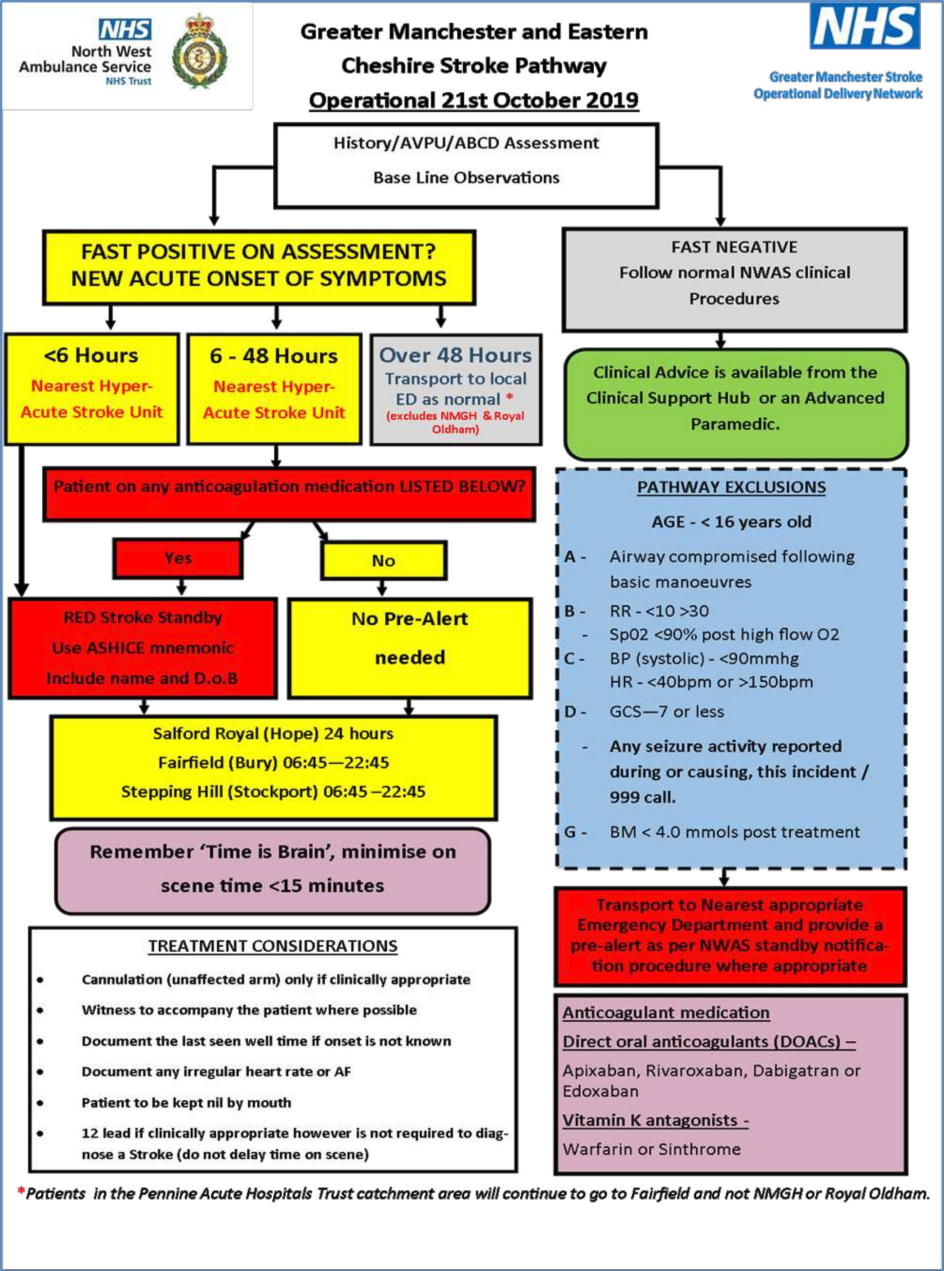
Greater Manchester (GM) stroke pathway for patients suspected with stroke. AVPU, (Alert, Voice, Pain, Unresponsive); ABCD, (Airway, Breathing, Circulation, Disability); ED, emergency department; ASHICE, (Age, Sex, History, Injuries/Findings, Condition, Estimated Time of Arrival); AF, atrial fibrillation; RR, respiratory rate; BP, blood pressure; HR, heart rate; GCS, Glasgow coma scale; BM, blood glucose; NMGH, North Manchester General Hospital; NWAS, North West Ambulance Service; NHS, National Health Service.

Since acute stroke care is increasingly centralised at specialist facilities, not all hospitals are equipped to provide acute stroke treatments; hence, correct destination depends on accurate prehospital diagnosis.^6^ For example, patients with LVO should be transported directly to a Comprehensive Stroke Centre (CSC) for thrombectomy treatment. Candidates for thrombolysis, meanwhile, can be taken to either a CSC or a Primary Stroke Centre (PSC) to receive treatment within the optimal timeframe. In contrast, patients with stroke mimics, stroke onset beyond 48 hours, or those meeting pathway exclusion criteria should be transported to the nearest emergency department (ED). Figure 1 illustrates the current Greater Manchester (GM) stroke pathway for patients suspected with stroke.

A prehospital video triage (PHVT) system allowing real-time communication between prehospital clinicians and hospital stroke specialists may enhance diagnostic accuracy, improve adherence to prehospital pathways, speed up stroke treatment, and reduce the transfer of patients without stroke to specialist stroke centres.^15^,^18^ A PHVT pilot (duration=7 weeks and 4 days) was conducted at a UK CSC, accompanied by a mixed-method service evaluation.

## Design

The PHVT pilot aimed to improve prehospital stroke diagnosis, reduce unnecessary transfers of stroke mimics to the CSC, and identify stroke subgroups that require specialised treatment at specific hospitals, thus facilitating a faster treatment delivery within the timeframe. The service evaluation aimed to determine which aspects of the PHVT intervention were effective, what facilitators and barriers existed, and how PHVT could be improved for future implementation.

### Setting

In GM, there is one CSC at Salford Royal Hospital, two PSCs and 10 EDs within the stroke pathway catchment area. The CSC operates 24 hours a day, conducting around 5000 assessments annually and resulting in approximately 2200 stroke admissions. It serves a population of 3.1 million and is the only hyperacute stroke unit operating within the hours of 22:45 and 06:45, as well as the sole centre providing 24-hour MT for the entire GM population, accommodating up to 400 eligible patients annually. The PSCs provide IVT and admit patients suspected with stroke within 48 hours of symptom onset from 06:45 to 22:45, while patients presenting after 48 hours are directed to the nearest ED.

### The PHVT pilot

The pilot was conducted in collaboration with the North West Ambulance Service (NWAS) and prehospital clinicians serving the western sector of GM, from 24 October 2022 to 16 December 2022. Funding was provided by the National Health Service (NHS) England Stroke Programme as part of a programme of national PHVT pilots in England.

#### The intervention—PHVT

Due to restricted staff availability at the CSC, PHVT was offered only during office hours (09:00–17:00, Monday–Friday). NWAS staff were asked to undertake a PHVT call for all patients suspected with stroke during these hours when the CSC was the nearest hyperacute stroke unit. Outside of these hours and in cases where PHVT was not used or was unavailable for technical reasons, the existing prehospital stroke pathway was followed. Stroke clinicians at the CSC volunteered to participate in a rota to take PHVT calls while not on clinical duty and received additional payment for their time. Stroke consultants covered most shifts but advanced care practitioners and middle-grade doctors also covered some shifts with consultant support.

A paper proforma was developed beforehand to structure the call and gather key clinical details (eg, Face, Arms, Speech, Time (FAST) assessment, onset time, and medical history) and call information (ambulance arrival time, nearest CSC and ED). A bespoke online database in an existing referral platform (PatientPass) was developed to capture data call completion and provide a clinical record. PHVT calls were made on iPads or iPhones with FaceTime. Over 80% of prehospital clinicians already had NWAS iPads, leveraging existing 4G and NWAS vehicle Wi-Fi capabilities. The final outcome of PHVT calls were as follows:

- Transfer to nearest ED: suspected non-stroke; pathway exclusion present; > 48 hours post onset; resolved symptoms.
- Transfer to CSC, immediate discussion with on-call team: suspected stroke <48 hours post onset; potential MT and/or IVT candidate.

#### Training

Before the pilot, stroke clinicians underwent online training with the PHVT project team via Teams, which was also recorded for those unable to attend. Training included a specific project training video demonstrating the PHVT process, patient assessment and conveyance decision-making, which was also made available to NWAS staff. NWAS publicised the pilot through their bulletins and emails, including details of the proposed PHVT call procedure.

### The service evaluation

We undertook a mixed-method service evaluation. All key care processes, diagnoses, outcomes and treatments were captured for patients undergoing PHVT during the pilot period. We undertook semistructured interviews with ambulance crews, stroke clinicians taking calls and patients/carers with personal experience of the PHVT process during the pilot.

#### Quantitative evaluation

##### Data collection and confidentiality

We identified patients who underwent PHVT through the PatientPass database, with stroke clinicians confirming final diagnosis using existing clinical records. Data describing all stroke patients admitted to the CSC during the PHVT pilot dates were extracted from the Sentinel Stroke National Audit Programme database at the CSC. All data were securely stored on the NHS network. Patient-identifiable data were managed exclusively by the local care team.

##### Statistical analysis

Prior to the study, we estimated that an average of 12 patients per week would be eligible for PHVT during office hours, resulting in an expected total of 96 assessments over the course of the pilot. This estimate was derived from local data on confirmed stroke arrivals over a 10-week period and was used to approximate ambulance arrivals within the hours of 09:00–17:00 Monday–Friday.

Given the small expected number during the PHVT pilot project, we lacked statistical power to test whether PHVT significantly altered diagnostic accuracy or process measures. We thus present descriptive statistics describing diagnosis, prehospital care processes and hospital care processes for patients undergoing PHVT. Data are presented as median and IQRs, numbers and percentages along with an exploratory analysis to assess differences in the characteristics of PHVT patients, in addition to relevant process measures and timings between PHVT patients and non-PHVT patients.

### Qualitative evaluation

#### Design and setting

We conducted one-time semistructured interviews with stakeholders involved in the PHVT pilot to understand the perceived utility, barriers and facilitators.

#### Sample and data collection

Our sample included 12 hospital clinicians (eight consultants, two registrars and two advanced practitioners) who undertook PHVT shifts, seven prehospital clinicians (five senior paramedics and two paramedics) who made PHVT calls and seven patients (and four of their family members) who underwent PHVT. Most patient participants (n=6) were transported to the CSC (and were diagnosed with stroke/TIA), and one was admitted to their local ED (diagnosed with a non-stroke condition).

Data collection took place between November 2022 and May 2023. We undertook 26 interviews: 21 online interviews; four telephone interviews and one face-to-face interview. Interviews lasted for an average of 34 min. Interviews were recorded digitally on a university computer using Teams software or on an encrypted audio device. Verbal informed consent was recorded on an encrypted audio device ahead of remote interviews and written informed consent was taken ahead of the face-to-face interview.

#### Data analysis

We undertook thematic analysis, known for its flexibility and adaptability to exploring qualitative data.^19^ The process involves data preparation, thematic coding, theme interpretation and data reporting.^20 21^

In data preparation, audio recordings of interviews were transcribed verbatim by a university-approved transcription company. Transcripts were checked for accuracy and deidentified. Data were managed using NVivo V.12,^22^ a qualitative data analysis software program. We then began the process of thematic coding by identifying initial themes and refining them. We interpreted these themes to identify complex relationships and patterns beyond individual themes to gain a better understanding of how they interacted with each other and to provide a more holistic picture. Lastly, we summarised the main findings from the data.

## Result

### Quantitative evaluation

**Figure 2:**
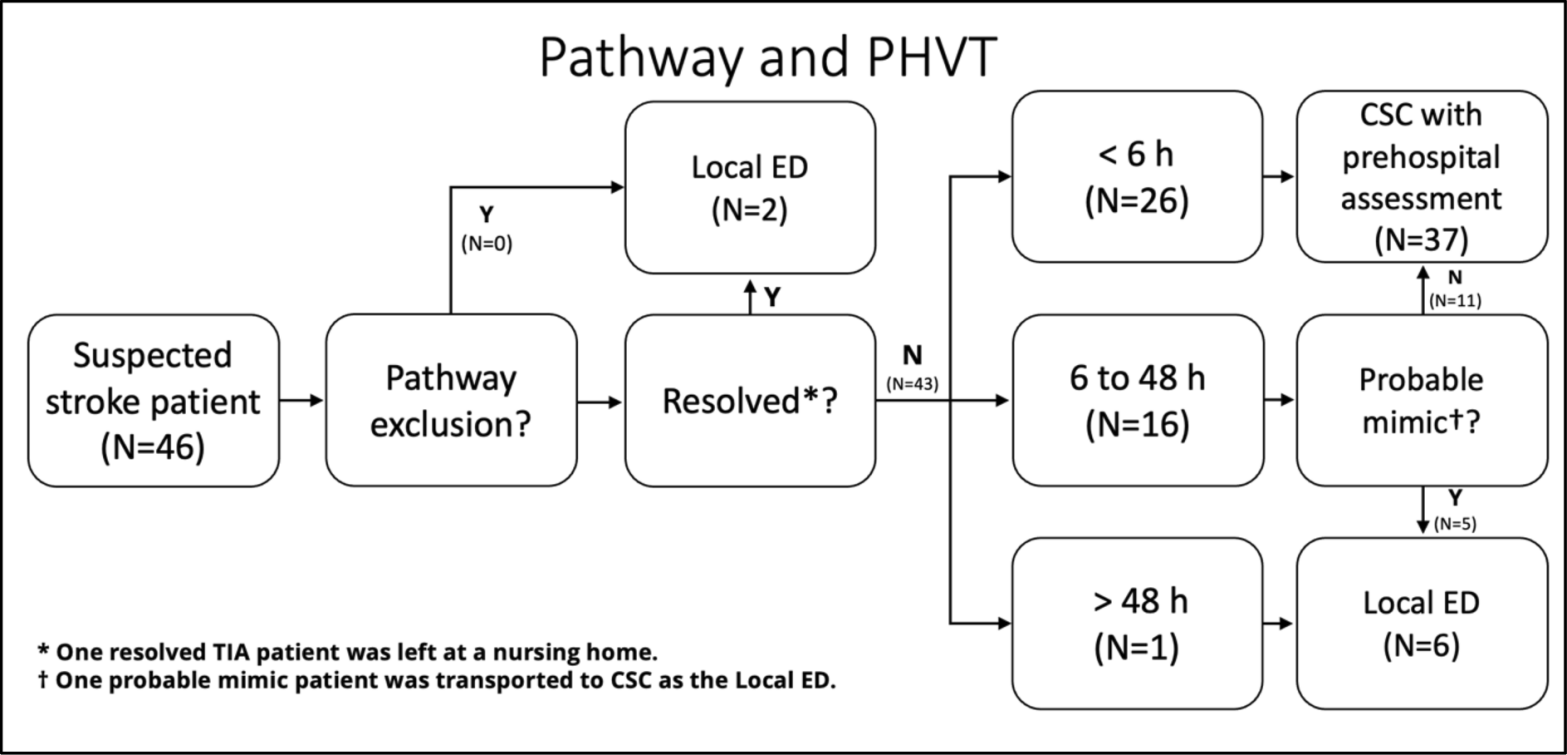
The stroke pathway and prehospital video triage (PHVT) patient’s destinations. CSC. Comprehensive Stroke Centre; ED, emergency department.

In total, 46 PHVT calls were made during the pilot. Hospital clinicians worked 1–4 shifts each and received a median of 2 calls per shift (range 0–5). During the study period, there were 84 confirmed patients who had a stroke admitted to the CSC during PHVT hours, of which only 26 (31%) had a PHVT call. Figure 2 demonstrates the pathways and destinations of PHVT patients, with 37 (80%) taken to the CSC. Of these, the final diagnosis was stroke in 14 (38%), TIA in eight (22%) and other diagnoses in 15 (40%). Of the eight (18%) patients transferred to the nearest ED, five (63%) were non-strokes, two (25%) were TIAs and one (12%) had a stroke but was >48 hours post onset, making the local ED the correct destination. There was only one patient (2%) left at their usual residence, since the patient was in a nursing home and had a fully resolved TIA.

#### Diagnostic accuracy

During 40 (87%) PHVT calls, the stroke clinician recorded stroke/TIA as the provisional diagnosis, with other diagnoses suspected in six (13%) calls. Table 1 describes the characteristics of PHVT call patients by final diagnosis. Overall, 14 (35%) suspected strokes had other non-stroke final diagnoses (false positives) but there were no false negatives, giving a sensitivity of 100% (95% CI 86.8% to 100%) and a specificity of 30% (95% CI 11.9% to 54%). LVO was suspected in seven cases and this was confirmed in three of these cases (43%). Six of the seven patients suspected with LVO (86%) had a final diagnosis of stroke. There were no false negatives, indicating a true negative rate of 100%.

**Table 1:** Characteristics of patients undergoing PHVT assessment by final discharge diagnosis

	Level	Stroke/TIA (n=26)	Other diagnosis (n=20)	P value*
Age (years)	Median (IQR)	81 (74–90)	78 (68–84)	0.13
Sex	Male, n (%)	12 (46%)	8 (40%)	0.76
Prestroke mRS	0–2 n (%)	15 (58%)	12 (60%)	0.75
	3–5 n (%)	10 (38%)	6 (30%)	
	Missing	1 (4%)	2 (10%)	
GCS	Median (IQR)	14 (14–15)	15 (14–15)	0.66
Blood glucose (mmol/L)	Median (IQR)	7.2 (6.3–9.4)	6.7 (6.1–8.8)	0.21
Systolic BP (mm Hg)	Median (IQR)	144 (132–176)	153 (136–174)	0.79
Diastolic BP (mm Hg)	Median (IQR)	80 (71–98)	84.5 (74–96)	0.67
Pulse (beats per minute)	Median (IQR)	72 (68–81)	88 (70–96)	0.054
Temperature	Median (IQR)	36.7 (36.4–37.0)	36.5 (35.9–36.7)	0.25
Onset to PHVT call (min)	Median (IQR)	129 (75–157)	56 (38–131)	0.065
Length of PHVT call (min)	Median (IQR)	8 (6–9)	8 (6–11)	0.60
Anticoagulation	Yes (%)	6 (23%)	6 (30%)	0.74
History of atrial fibrillation	Yes (%)	4 (15%)	3 (15%)	1.00
FAST test	Positive, n (%)	22 (85%)	13 (65%)	0.17
Symptoms				
Face weakness	Yes, n (%)	13 (50%)	7 (35%)	0.36
Arm weakness	Yes, n (%)	12 (46%)	11 (55%)	0.44
Speech problem	Yes, n (%)	18 (69%)	16 (80%)	0.91
Leg weakness	Yes, n (%)	9 (35%)	7 (35%)	0.66
Destination after PHVT	CSC; n (%)	22 (85%)	15 (75%)	0.43
	Local ED; n (%)	3 (12%)	5 (5%)	
	Home; n (%)	1 (3%)	0 (0%)	

* The Mann-Whitney U-test was performed for continuous variables, and Fisher’s exact test was performed for categorical variables.

CSCComprehensive Stroke CentreEDemergency departmentFASTFace, Arms, Speech, TimeGCSGlasgow Coma ScalemRSmodified Rankin ScalePHVTprehospital video triage

#### Process measures

Median time from arrival on scene to PHVT call was 26 min (range 6–67), call duration was 8 min (range 4–28) and the median time from ending the call to leaving the scene was 2 min (IQR 0–3). Median time on scene was 41 min (IQR 32–47). Median time from symptom onset to arrival at hospital was 170 min (IQR 115–359). Fallout of the PHVT calls was experienced on five (11%) occasions, poor video was experienced in two (4%) and poor audio in two (4%) other calls.

We compared all patients who had a stroke admitted to the CSC while the PHVT service was running, dividing them into those who had a PHVT call and those who did not (table 2). Onset-to-arrival and arriving within 4 hours of onset were significantly different (p value<0.001). The wait times for PHVT patients who had a stroke to see a consultant and nurse at CSC were lower (38 vs 105 min and 48 vs 63 min, respectively), though not statistically significant (p values 0.14 and 0.99, respectively). While PHVT patients had slightly longer times for arrival to scan (38 vs 27 min), swallow assessment (113 vs 73 min) and IVT (52 vs 37 min), again, these were not statistically significant (p values 0.80, 0.051 and 0.42, respectively).

**Table 2:** The characteristics and care process measures of patients who had a stroke admitted during PHVT’s operational period, divided by those that received a PHVT call versus no PHVT call made

	Level	PHVT patients who had a stroke in SSNAP(n=14)*	CSC SSNAP dataset for patients not in PHVT (09:00–17:00 ambulance arrivals only)(n=84)	P value†
Age (years)	Median (IQR)	80.5 (61–87)	79 (67–85)	0.80
Sex	Male, n (%)	10 (71%)	47 (56%)	0.38
Pre-stroke mRS	0–2 n (%)	8 (57%)	58 (69%)	0.47
	3–5 n (%)	6 (43%)	26 (31%)	
Onset to arrival(min)	Median (IQR)	170 (115–359)	970 (162–1038)	<0.01
Arriving within 4 hours of onset	(%)	71.40%	31%	<0.01
Onset to arrival within 4 hours(min)	Median (IQR)	126(96–171)	135(108–180)	0.91
Total NIHSS on arrival	Median (IQR)	8.5 (6–18)	6 (4–14)	0.32
Time to see consultant(min)	Median (IQR)	38(25–110)	105(20–206)	0.14
Time to see nurse(min)	Median (IQR)	48(13–181)	63(13–156)	0.99
Imaging—arrival to scan(min)	Median (IQR)	38(7–63)	27(14–94)	0.80
Arrival to swallow assessment(min)	Median (IQR)	173(126–226)	113(51–207)	0.051
Stroke type	IS, n (%)ICH, n (%)	13 (93%)1 (7%)	74 (88%)10 (12%)	1.00
Arrival to start of blood pressure lowering(min)	Median (IQR)	23(n/a)	34(24–98)	n/a
Anticoagulant	Yes n (%)	3 (21.4%)	26 (31%)	0.54
Arrival to reversal of anticoagulation (min)	Median (IQR)	n/a	107 (n/a)	n/a
IVT	n (%)	3 (21.4%)	12 (14.3%)	0.44
Arrival to tPA (min)	Median (IQR)	52 (n/a)	37 (34–84)	0.42
IAT	n (%)	1 (7.0%)	6 (7.0%)	1.0
Door to puncture (min)	Median (IQR)	110 (n/a)	144 (64–131)	n/a

* Of the 26 patients who had a stroke/TIA final diagnosis, 15 patients had a stroke; 14 patients were transported to CSC and appeared in SSNAP, and one patient was transported to local ED and did not appear in SSNAP.

† The Mann-Whitney U-test was performed for continuous variables, and Fisher’s exact test was performed for categorical variables.

CSCComprehensive Stroke CentreEDemergency departmentIATintra-arterial therapyICHintracerebral haemorrhageISischaemic strokeIVTintravenous thrombolysismRSmodified Rankin ScaleNIHSSNational Institutes of Health Stroke ScaleSSNAPSentinel Stroke National Audit ProgrammetPAtissue plasminogen activator

### Qualitative evaluation

We present three overarching themes, supported by illustrative quotes.

#### Perceived value of PHVT for suspected stroke patients

There were mixed views about the value of PHVT among participants. Most hospital clinicians perceived PHVT positively, highlighting its potential to identify and filter patients without stroke, thereby reducing the CSC burden.

I felt that this…was required because we tend to see a lot of patients who…may not need to come [to CSC] if we had an out of hospital triaging…method. So that’s why I was quite excited about it. Stroke Consultant 7

Similarly, most paramedics viewed PHVT positively, valuing help in making informed decisions in complex cases and appreciating expert guidance for accurate diagnosis and appropriate hospital transfer. Paramedics did report initial scepticism towards the need for PHVT, feeling confident in their ability to identify FAST-positive patients, although these concerns were allayed once they had used the service.

… at first, I was very doubtful. I was thinking, I don’t need someone to tell me if this patient is FAST positive or not…I can do that assessment, I don’t need to video someone… Senior Paramedic 1

Most patients/carers valued the paramedics’ ability to consult specialists through PHVT, finding reassurance in speaking with specialists about their condition and being transferred to the appropriate hospital for treatment.

… it’s better for them to know which hospital to take you to, it cuts down the time…to get you to the right hospital. Patient 2

In contrast, some hospital clinicians questioned the feasibility of remote assessments to enable them to divert patients away from CSC based on video or telephone consultations.

… I didn’t feel that clinicians would be willing to take a risk, based on a telephone consultation or telemedicine consultation from a person who’s a non-clinician on the other side, a paramedic…. Stroke Consultant 6

In addition, they reported similar attitudes among prehospital clinicians who had arrived in ED without using PHVT for patients suspected with stroke. They reported prehospital clinicians’ reluctance to use PHVT, preferring not to prolong on-scene time, especially when dealing with FAST-positive patients likely to be transferred to CSC regardless. They also reported low awareness of the PHVT pilot among prehospital clinicians, highlighting the need to raise awareness and use of PHVT.

People who were bringing patients in but hadn’t used video triage and just sort of telling me why they’d not used video triage because they were so certain that the patient needed to come anyway so they’d not called us before. Stroke Consultant 9

Patients/carers also reported how prehospital clinicians did not seem satisfied with PHVT, commenting to patients that it was an unnecessary process.

… I don’t think they were happy about it… Because they were basically saying, oh, well, we know what is wrong with you, I have been doing this job for 30 years, I don’t know why we have got to do this consultation… Patient 4

#### Ability to divert stroke mimics via PHVT

Before the PHVT pilot, hospital clinicians reported anticipating a reduction in stroke mimic transfers to CSC but some felt this goal was not fully achieved. They encountered challenges during video examinations that hindered their confidence in accurately identifying and diverting all stroke mimics as hoped.

…we’re not as brave and as clever as we think in ourselves to be in [ED] when you’ve got the full picture. Stroke Consultant 5

Hospital clinicians felt that PHVT could not replace in-person ED assessments, as it often missed nuanced neurological symptoms. They noted that direct patient interactions in ED revealed subtle signs which were difficult to detect on video, often due to call quality or limitations in patient positioning during PHVT examinations.

It’s quite different examining someone on a video call with an iPad…So you could think, for example, that they might have a facial droop on a video because of their position…But when you have them in front of you …it might just be that it’s not true. Stroke Consultant 2

Some pointed out difficulties with PHVT assessments due to how prehospital clinicians managed the iPad. They observed inadequate views when the camera was either held too far, only showing the patient’s face, or positioned too closely, hindering a comprehensive assessment when asked to view other parts of the patient. This prompted hospital clinicians to suggest future PHVT training for prehospital clinicians, focusing on maintaining optimal camera views during PHVT procedures. Technical issues, including poor connections, voice and/or video disruptions and complete call drops, further hindered assessments. Hospital clinicians reported that sometimes they could not see or hear patients, impacting assessment and information gathering. Despite technical challenges, hospital clinicians found the PHVT software easy to navigate. In contrast, most paramedics experienced difficulties, particularly with initial setup and login, due to their limited experience with iPads and video calls.

… the iPads were new that we were using, we hadn’t had them long. So, when it came to do the triage, I wasn’t signed into my Apple ID, so I couldn’t use FaceTime, things like that. So that was the only thing that hindered it initially… Paramedic 5

Overall, the majority of hospital clinicians, paramedics and patients/carers found the sound and picture quality to be satisfactory, facilitating communication and examinations.

…it was just as if she was in the room… very clear. A very clear picture, just like I was speaking to her in the room. Patient 2

However, a few hospital clinicians felt that the quality was insufficient to conduct a proper clinical assessment, suggesting a need for higher-tech solutions.

The quality of the picture and the sound [is] not good enough to examine a patient thoroughly and make a medical decision on that. Stroke Consultant 2

#### Changes in working practices/patient’s outcome

Opinions varied on PHVT’s perceived impact on work practices. Some hospital clinicians noted improvements, such as receiving more detailed patient information, which aided on-call teams in better preparation and ensured thorough handovers, particularly when there was no direct interaction with prehospital clinicians.

The most helpful aspect of it was getting the history from the ambulance crew faster than getting the history in the ED department. So I could then pass along that information to the on-call team where decisions were pre-emptively made already. Stroke Registrar 10

Paramedics stated that the handover process in ED was expedited for PHVT patients because the stroke specialists greeting them had more comprehensive prearrival patient information. They also reported increased confidence in managing patients suspected with stroke when using PHVT, valuing specialist guidance in ambiguous cases for better conveyance decisions.

I saw quite a remarkable difference… and this instance of patient care I thought that actually we’ve escalated everything, and everything’s happened a little bit quicker because we’ve been able to speak to the consultant en route… Senior Paramedic 1

Some hospital clinicians believed PHVT improved relationships and communication with prehospital clinicians, seeing it as an opportunity for them to learn stroke assessment techniques, potentially supporting future conveyance decisions for hyperacute treatment cases. Others felt it fostered collaboration, emphasising the pilot’s goal of enhancing patient care rather than questioning prehospital clinicians’ knowledge.

… we were able to get them to think a little bit differently as well, as to whether the patient was appropriate for the stroke pathway or not…I felt it helped the communication. Advanced Care Practitioner 1

Conversely, some hospital clinicians felt that PHVT had little impact, noting no significant change in patient preparation processes or added value. These were concerned that PHVT might introduce delays for hyperacute patients. Several hospital clinicians and paramedics felt that PHVT did not result in significant changes in their work practices or professional relationships, which may be due to their limited experience of using PHVT.

I’m not sure whether me being on the phone really added anything to that experience of the patient. It may have delayed, actually, the treatment because there was an extra step where they had to call me and then I had to confirm and then they would bring the patient into the same place they were going to bring anyway. Stroke Consultant 3

Most patients/carers saw PHVT as a positive influence on their care experience. They valued the presence of an informed stroke team on hospital arrival, which they felt shortened waiting times and expedited admissions and treatment. Patients who had a previous stroke, and were transported to the hospital without PHVT previously, found this a significant change.

They said, we’ve been in contact with [CSC] and they’re waiting for us…Within two and a half hours of the event, I guess she was having the emergency brain surgery… I couldn’t imagine this had gone any faster or any better and we are both indebted to that team. Carer 1

However, a small number of patients believed PHVT caused delays in their care, which they attributed to technical issues and prolonged on-scene PHVT initiation. Others believe that it needed more buy-in from paramedics to work effectively.

I think they were more concerned of setting this pilot scheme up, the video than doing what they should have done naturally and just got her to hospital. Carer 3

I think from a holistic point of view, if you had the buy in from the paramedics your patients might feel as if this is a really worthwhile thing to do. Patient 4

Participants' views on PHVT’s impact on patient outcomes varied. Some hospital clinicians and paramedics felt that PHVT positively influenced patient outcomes by expediting timely interventions, such as door-to-needle procedures resulting in improved outcomes. Also, they believed that by directing patients to the most suitable treatment facility, PHVT reduced unnecessary conveyances, avoiding prolonged waiting and subsequent transfers. Patients/carers believed that PHVT helped them get to the most appropriate hospital promptly, resulting in a better outcome and care.

For example, our [patient]… went home after 24–48 hours of having thrombolysis and IAT in the centre and …is functioning. So, I think that…is a huge impact. Stroke Consultant 1

Cause without that he could have been taken to [local hospital]… And it’s such a simple and easy thing to do really… within at least an hour and a half…they’d started the clot buster. So, you know, from that point of view it was really good. Carer 5

However, some hospital clinicians argued that PHVT’s influence on patient outcomes was negligible. They indicated that PHVT might prolong door-to-needle times for hyperacute cases if it caused prolonged on-scene time. Others believed PHVT did not change conveyance decisions, often maintaining pre-PHVT decisions. Participants expressed needing more experience with PHVT to meaningfully impact patient outcomes.

…for hyperacute treatments it may have increased the time period a little bit, but for the rest of the amber patients, it’s probably been it wouldn’t have really changed anything too much. Stroke Consultant 3

Participants suggested the need for system changes to ensure successful implementation of PHVT to improve work practices and patient outcomes. These involved enabling patients to be registered on to the hospital system ahead of arrival, allowing clinicians to preorder scans, and having 24-hour PHVT service availability.

I think unless… you can preregister the patients before they come in… Only then I see it being effective… The patient needs to be registered on EPR and the CT scan should have been booked, and the CT scanner should be ready for the patient as soon as they come in. Stroke Consultant 6

Others questioned the PHVT pilot’s rota-based approach, proposing that on-call teams were better placed to handle PHVT calls to streamline the process. Some also suggested implementing a screening process to manage high patient volumes, whereby nurses could triage calls, with consultant input as needed. This was seen as vital for effective PHVT use in a busy stroke centre.

I think it should be a screening process and then if it’s the case where it fulfils the criteria then you involve a consultant… And that could be somebody who’s already on call because you have to be on the shop floor. Stroke Consultant 1

## Discussion

### Summary of the findings

Despite the lower-than-expected volume of PHVT calls compared with the number of patients who arrived at the CSC without PHVT, there was a 20% reduction in patients without stroke transported to the CSC, alleviating some burden. The PHVT’s identification of LVO enhanced hospital readiness, as perceived in some of the qualitative findings. Within the PHVT cohort, only one patient received IAT, achieving a shorter time to treatment compared with non-PHVT patients; however, this result was not statistically significant. The data further showed that PHVT patients had significantly reduced times for arrival at the hospital within 4 hours of onset and for the start of stroke onset to hospital arrival timing.

However, the overall impact of PHVT on other patient timelines presents a nuanced picture. While PHVT patients received quicker reviews from stroke consultants and nurses on arrival, and faster blood pressure management where necessary, they also experienced longer times for procedures such as scanning and swallow assessments, and IVT treatment.

This is somewhat at odds with qualitative insights, where data suggested that PHVT patients would benefit from expedited handovers and the presence of additional staff on arrival, facilitating prompt access to further assessment and treatment. These mixed findings may, therefore, reflect the constraints of the limited sample size, which complicates drawing robust inferences about PHVT’s impact on all treatment timelines.

Sixty-two per cent of CSC transports were ultimately non-stroke diagnoses. This may stem from the CSC being the closest ED for some, combined with challenges in diverting non-stroke cases via PHVT. In addition, paramedics’ limited proficiency in using the PHVT effectively was a key challenge, highlighting the need for focused training to mitigate this issue. The absence of formal training for prehospital clinicians prior to the pilot is regarded as a primary factor contributing to stroke clinicians’ difficulty conducting comprehensive assessments to divert all non-stroke cases. This lack of training resulted in inconsistencies in PHVT usage and may have introduced delays in patient assessments, as clinicians needed additional time to navigate the unfamiliar tool. These training gaps particularly impacted the effective use of video technology, with issues such as suboptimal camera positioning during patient assessments being reported. Furthermore, technical challenges, including limited familiarity with the software, hindered the operation of the PHVT system.

Clinicians’ responses to PHVT were also varied. Hospital staff acknowledged its potential for improved preparedness and patient outcomes, while paramedics reported increased confidence in stroke management. Conversely, some describe no significant change in practices or outcomes, citing the need for greater awareness and usage among prehospital clinicians. Hospital clinicians suggested prearrival registration of patients, which could streamline admissions and treatments, offsetting PHVT-related delays. Currently, even if the hospital clinicians confirm the patient requires a specific treatment or scan, this cannot be done until they arrive at the hospital.

Despite delays on the scene caused by technical difficulties in some cases, patients/carers consider PHVT beneficial, but connectivity, on-scene delays, and clinicians' buy-in must be considered.

### Comparison with previous literature

Hospital clinicians’ perception that PHVT improved patient care preparation aligns with Ramsay *et al*,^23^ who reported that preassessment via video allowed stroke teams to be better prepared on patient arrival. Similarly, paramedics’ increased confidence in identifying strokes in challenging cases echoes findings by Brunton *et al*,^24^ highlighting ambulance personnel’s previous uncertainty in stroke identification. This boost in confidence is further supported by Ramsay *et al*,^23^ noting enhanced reassurance in patient transport decisions with PHVT.

The need for paramedic training, as suggested by hospital clinicians, is corroborated by studies emphasising the importance of training for effective PHVT assessments,^23 25 26^ demonstrating that training is a critical component for the effective use of PHVT and similar technologies. Technical challenges, like connectivity issues affecting assessments, are also noted in previous research.^23 26^

During the pilot, hospital stroke clinicians, working additional compensated shifts, handled PHVT calls separately from their routine clinical duties. However, for future improvements, they suggested involving the on-call team in handling the calls and nursing staff in filtering PHVT calls. This contrasts with Ramsay *et al*,^23^ where clinicians faced challenges in managing PHVT calls alongside ward duties. Additionally, ambulance clinicians perceived direct PHVT calls to stroke physicians, bypassing stroke nurses, to be beneficial for reducing delays.

### Limitations

Our study faced limitations. Limitations of the pilot included the short duration of the pilot, as well as the low call volume. This restricted clinicians’ exposure to the PHVT system, which possibly influenced their views and limited their experience. The lack of comprehensive training for prehospital staff hindered patient assessments via video triage. Limitations of the evaluation included the recruitment of a limited number of prehospital clinicians, exclusively paramedics, which narrowed thorough insight and missed potentially diverse perspectives from emergency medical technicians. Patient interviews were limited and mainly from those transported to the CSC. A broader sample, including patients diverted by PHVT, might offer a more comprehensive evaluation of PHVT’s impact.

Lastly, our initial ambition was to link prehospital and hospital data for all suspected stroke cases gathered by NWAS in GM western sector. However, due to significant information governance barriers, we were unable to proceed with this within the time frame and resources of the project.

### Implications for practice/policy

The PHVT pilot demonstrates the potential to enhance stroke care by facilitating interventions, reducing unnecessary transfers and improving patient outcomes. However, for its benefits to be fully realised and drawbacks minimised, improvements are essential. A longer duration pilot with a larger number of cases is necessary to fully assess PHVT benefits. Comprehensive training for both hospital and prehospital clinicians is critical to system success. Securing adequate funding, raising awareness and providing thorough training with educational feedback are essential to maximise PHVT’s impact. As key stakeholders in the adoption and success of this new pathway, prehospital clinicians need to be well versed in both the technology and assessment protocols to enhance patient triage timeliness and accuracy.

Technical improvements are also necessary, particularly addressing connectivity issues and investing in infrastructure to prevent technical difficulties that could delay assessments and impact patient care. Additionally, modifying hospital systems to allow prearrival patient registration could streamline processes. This change would enable quicker preparation and treatment for arriving patients, enhancing overall patient care and outcomes.

## Conclusion

Stroke remains one of the leading causes of mortality and disability in the world, and PHVT can assist in identifying a suitable hospital accurately and quickly, thereby improving patient outcomes. Although the number of patients during the pilot was limited, PHVT reduced the transportation of some patients without stroke to CSC and expedited some stroke care measurements.

Most hospital clinicians, paramedics and patients expressed satisfaction with PHVT’s sound and video quality. While hospital clinicians found the technology intuitive, paramedics faced challenges due to unfamiliarity with the software. Both paramedics and hospital clinicians recognised PHVT’s utility in selecting the most suitable hospital and in preparing for patients' arrival. The majority of patients felt their care quality improved with PHVT. However, to enhance effectiveness and mitigate risks, participants recommended further improvements.

## Data Availability

Data are available upon reasonable request.

## References

[1] National Audit Office (2005). Reducing brain damage: faster access to better stroke care.

[2] Price CI, Shaw L, Dodd P (2019). Paramedic Acute Stroke Treatment Assessment (PASTA): study protocol for a randomised controlled trial. Trials.

[3] Wardlaw JM, Murray V, Berge E (2014). Thrombolysis for acute ischaemic stroke. Cochrane Database Syst Rev.

[4] National Institute for Health and Care Excellence (2012). Alteplase for treating acute ischaemic stroke (review of technology appraisal guidance 122).

[5] Saver JL, Goyal M, van der Lugt A (2016). Time to Treatment With Endovascular Thrombectomy and Outcomes From Ischemic Stroke: A Meta-analysis. JAMA.

[6] Trialists’Collaboration SU (2013). Organised inpatient (stroke unit) care for stroke. Cochrane Database Syst Rev.

[7] Hacke W, Kaste M, Bluhmki E (2008). Thrombolysis with Alteplase 3 to 4.5 Hours after Acute Ischemic Stroke. N Engl J Med.

[8] Albers GW, Marks MP, Kemp S (2018). Thrombectomy for Stroke at 6 to 16 Hours with Selection by Perfusion Imaging. N Engl J Med.

[9] Evans MRB, White P, Cowley P (2017). Revolution in acute ischaemic stroke care: a practical guide to mechanical thrombectomy. Pract Neurol.

[10] Emberson J, Lees KR, Lyden P (2014). Effect of treatment delay, age, and stroke severity on the effects of intravenous thrombolysis with alteplase for acute ischaemic stroke: a meta-analysis of individual patient data from randomised trials. The Lancet.

[11] Campbell BCV, Mitchell PJ, Kleinig TJ (2015). Endovascular therapy for ischemic stroke with perfusion-imaging selection. N Engl J Med.

[12] Harbison J, Hossain O, Jenkinson D (2003). Diagnostic accuracy of stroke referrals from primary care, emergency room physicians, and ambulance staff using the face arm speech test. Stroke.

[13] Gibson LM, Whiteley W (2013). The differential diagnosis of suspected stroke: a systematic review. J R Coll Physicians Edinb.

[14] Rudd M, Buck D, Ford GA (2016). A systematic review of stroke recognition instruments in hospital and prehospital settings. Emerg Med J.

[15] Johansson A, Esbjörnsson M, Nordqvist P (2019). Technical feasibility and ambulance nurses’ view of a digital telemedicine system in pre-hospital stroke care – A pilot study. Int Emerg Nurs.

[16] LaMonte MP (1998). Tele-BAT: mobile telemedicine for the Brain Attack Team. Stroke.

[17] Bergrath S, Reich A, Rossaint R (2012). Feasibility of Prehospital Teleconsultation in Acute Stroke – A Pilot Study in Clinical Routine. PLoS One.

[18] Barrett KM, Pizzi MA, Kesari V (2017). Ambulance-based assessment of NIH Stroke Scale with telemedicine: A feasibility pilot study. J Telemed Telecare.

[19] Braun V, Clarke V (2006). Using thematic analysis in psychology. Qual Res Psychol.

[20] Guest G, MacQueen KM, Namey EE (2011). Applied thematic analysis.

[21] Boyatzis RE (1998). Transforming qualitative information: thematic analysis and code development.

[22] Ltd QIP (2020). NVivo (released in March 2020).

[23] Ramsay AI, Ledger J, Tomini SM (2022). Prehospital video triage of potential stroke patients in North Central London and East Kent: rapid mixed-methods service evaluation. *Health Soc Care Deliv Res*.

[24] Brunton L, Boaden R, Knowles S (2019). Pre-hospital stroke recognition in a UK centralised stroke system: a qualitative evaluation of current practice. *Br Paramed J*.

[25] Lumley HA, Flynn D, Shaw L (2020). A scoping review of pre-hospital technology to assist ambulance personnel with patient diagnosis or stratification during the emergency assessment of suspected stroke. BMC Emerg Med.

[26] Rogers H, Chalil Madathil K, Joseph A (2021). Task, usability, and error analyses of ambulance-based telemedicine for stroke care. IISE Trans Healthc Syst Eng.

